# Dr. Arnold, on Typhus, Dysentery, and Scurvy

**Published:** 1809-01

**Authors:** Joseph Arnold


### *To the Editors of the Medical and Physical Journal.*

GENTLEMEN,

IF the following observations on a much disputed subject should be found worthy of publication, I shall be glad to see them in your excellent Journal; they are very cursory, but the subject seems to deserve more attention from the medical public than I can at present give to it; and if any of your correspondents should think it worth while to answer any queries that may be started, I shall be extremely honoured by their observation; or should they wish to controvert any statement in my short dissertation, I trust I am not too obstinate to give up any thing that may appear ill founded: but if I should still think it necessary to retain my opinion, I will endeavour to give my reasons for it in a future letter.

The diseases to which I would now turn your attention, are *Typhus Fever, Dysentery,* and *Scurvy,* and my remarks will principally relate to the *causes, diagnosis,* and *method of cure* of these diseases, and particularly I shall make a few remarks on some cases in which the two contagious diseases were complicated with the scorbutic diathesis.

On the principal *exciting cause* of typhus fever, I believe, there can be no doubt, it is the first remark that is made in the very definition of the disease by our celebrated Nosologist, viz. "Morbus est contagiosus;" contagion therefore, if not the sole, is certainly the most common cause of this complaint; and although it may be difficult to discover the origin of this contagious matter, yet whenever we observe a well marked case of the disease, we certainly can have *no doubt* of its being contagious, and hence ought to guard against its spreading.

I scarcely think it worth while to enter into any argument with those persons who deny the contagious nature of typhus, as such a subject of dispute must terminate in endless contradiction, or perhaps, after all, might end only in a war of words; but I wish it to be understood, that typhus fever, in *whatever form it may appear, or with whatever disease it may be complicated, is uniformly contagious*; and why it does not always show itself so, depends upon another circumstance, viz. on the *prædisponent causes,* for every contagious disease is the effect of two causes, the exciting and the prædisponent.

With regard to the prædisponent causes of typhus, little can be said; the subject is very obscure, and in very many cases the prædisponent causes are altogether hidden from our knowledge; we see the strong and the weak, the corpulent and the thin, the old and the young, at times attacked by this complaint, we cannot, therefore, seek a prædisposition to the disease from these circumstances; some persons have believed, and have endeavoured to prove that *debility* is the prædisponent cause, but to this there are many strong objections. I recollect but one instance of a dropsical person taking the contagion of typhus, and that was from sleeping with a person labouring under that complaint; phthisical persons also, although very much exposed to the disease, seldom are affected by it; but here we have cases of the greatest debility, which yet do not prædispose to typhus.--Hence, then, debility received in its general acceptation, cannot be esteemed a prædisponent cause of typhus; but if we proceed in our investigation, and examine the various kinds of debility, we shall soon conclude, that certain modifications of it *do* become prædisponent causes; *that* debility, for instance, that arises from want of food, or from bad nourishment, induces a prædisposition to this complaint, for we daily observe that this complaint prevails most among the poor and incarcerated, and it seems to acquire virulence from this circumstance.—Other prædisponent causes are the depressing passions; and it is by some thought that certain states of the atmosphere have the effect of prædisposing to typhus.

The same remarks might be made with respect to dysentery, which prevails in camps, and on board ships, only when the men have been for a long time upon short allowance, bad food, or bad accommmodations.

I come now to the second part of my subject, which is to enumerate scurvy as a prædisponent cause of typhus and dysentery. This complaint is well known to be produced by want of proper nourishment, but especially of vegetable matter, by long continuance in the use of salted meat, by cold, inactivity, and the depressing passions; so that here we have the prædisponent cause of this disease running on to a complaint sui generis, and which only continues to be scorbutus as long as the exciting causes of typhus or dysentery are absent; when once these complaints appear, which they sometimes do, and proceed from combinations of circumstances which are often inscrutable to us, the havoc is dreadful; the scorbutic symptoms no longer characterize the complaint, the more dangerous typhoid attack attack the patient with ten-fold violence, and often in a few hours run on to his destruction; and here I must observe, that the symptoms of typhus and dysentery are altogether *foreign to scurvy*, and never would appear, unless the *exciting* causes of these diseases prevailed, so that it is a vast mistake to suppose that the symptoms of Typhus or Dysentery depend upon scurvy, and are only the more virulent symptoms of that disease; for scurvy often runs on to death without at all departing from its peculiar symptoms. Nor is the diagnosis between the two diseases at all difficult, for there are many circumstances which appear in the one entirely different from those of the other. The first approach of scurvy is known by a languid state of the body, by a bloated and pale countenance, by dejection of mind, and breathlessness on using the slightest exertion; in a short time the gums swell, become fungous and bleeding, the urine is high coloured, the breath fœtid, the teeth become loose, and the skin full of blotches, which at length run on to bleeding, fungous, and fœtid ulcers; the legs now begin to swell, the heart palpitates, and the patient on rising from his bed often faints, and sometimes even dies; at different periods of the complaint, the patient suffers from erratic pains like those of rheumatism, and often a cough comes on with pain of the side, so that some have been induced to bleed, supposing that the complaint was pneumonic; *during the whole course of the disease the pulse continues tolerably regular, and the appetite good, even till a few hours before death, and the intellects continue sound to the last.* But in typhus the appetite *instantly* fails, the *tongue becomes parched,* the *senses disturbed,* and the *skin* is *hot and dry,* while the *pulse is weak and quick, but variable,* so that in *all cases,* the last mentioned symptoms are sufficient to distinguish between the two diseases, and much more with respect to dysentery, where the frequent mucous and bloody alvine evacuations are sufficient alone to inform us of the complaint.

Having made these few remarks on the causes, the symptoms, and diagnostics of these three diseases, I shall proceed to mention some occurrences that lately came under my review, and which gave rise to these observations.

It was my fortune to be on board one of his Majesty's ships in the Baltic this summer, and the diseases in question prevailed on board the Swedish fleet; it was, I believe, officially announced, that the prevailing disease was scurvy, and as my principal reason for entering the service was observation, I could not but be very anxious to see a disease, which, perhaps, might never again be presented to me, for by the liberal allowance of lime juice, and other preventives of this once fatal complaint in the British navy, it will, perhaps, never again proceed to any dangerous extent.

It was announced to the English commander in chief, that the Swedish ships were in a most dangerous state from scurvy of *the * most inveterate and dangerous kind,* and that unless the ships were sent home, they would not have men sufficient to navigate them, that even the most *healthy part* of the companies had latent symptoms of † scurvy.

In consequence of such a report being made to the English commander in chief, five or six of the Swedish ships were sent home, and five line of battle ships only remained off Rogerwick. From curiosity alone, I went on board these five ships on the 21st of September, being accompanied by the surgeon of the Victory. I certainly expected to find men dying of scurvy, with scorbutic ulcers, œdematous legs, bloated faces, and frequent hæmorrhages; but instead of that, I saw persons labouring under hot and dry skin, parched tongue, quick and low pulse, staring eyes, stupor, muttering delirium, and picking the bed clothes; but still, although the symptoms so evidently marked the disease, I wished to hear the opinion of the Swedish surgeon on the cases; and as we could not converse in our vernacular tongues, we made use of the Latin in our conversation The first question I asked was, Quo morbo patiuntur hi ægrotantes? Answer, Typho nervoso. The next question I put was, An appetentia cibi deficit his ægris, morbo equidem incipiente? Answer, Etiam in toto. I put this interrogatory, because I think by the appetite alone, we may be able to distinguish between typhus and scurvy. The next question also was made with the same intention. An plurimi delirant? Answer, Plurimi. I demanded

* "It is now fully determined, that there is one disease only entitled to the appellation of scurvy; it is the same on land as at sea, and in all climates and seasons, depending nearly upon the same causes, and it is not at all diversified either in its phænomena or causes."—Vide Section 1790 of Dr. Cullen's Practice of Physic.

† It appears very strange, that a strong remonstrance was not made to the Swedish government, on the necessity of fresh provisions, for scurvy recovers as well at sea as on shore, when vegetable food is offered to the sick; and it must be recollected that all this time we were within two or three days sail of Stockholm, and it was at this time of the year when vegetables and fruits were in greatest plenty.

manded also the duration of some of their diseases, and found that those who were convalescent, had been ill a fortnight or three weeks; others, who were delirious, had complained only for a few days. I believe the name of the ship in which I made these remarks was the Dristigheten. There were about 20 men confined to their hammocks in this ship; the companies of the other ships appeared to be nearly in the same state; all of them were despondent, and some had evident marks of scurvy, such as sore gums, desire of solitude, nostalgia, and erratic pains; in one or two of the typhoid cases, I observed sore gums, apparently from scurvy having prevailed before the attack of typhus. I enquired after swelled legs, but saw none, and I saw but a few cases of dysentery, which were severe, and the patients delirious; but when the ships went into port, the latter seemed to be the prevailing disease, and was the death of several hundreds. In the admiral's ship, the Gustaf Adolph, were not more than twelve confined to their hammocks.

Having now mentioned the few observations that I made during a very short stay on board the Swedish ships, I cannot but express my surprise, that the principal disease prevailing there was reported to be scurvy; it certainly was very common among the ships, under the form of the scorbutic diathesis, and required immediate relief, as it gave a prædisposition to the more dangerous complaints, typhus and dysentery; but the *dangerous* complaints, and those that required the most immediate attention, were the two latter; but in none of the ships did I observe any means taken to prevent the spreading of the contagion; the typhoid, the dysenteric, the rheumatic, and all other sick persons, were confined together by a screen on the main deck, and I understand, that before the ships were visited by the English surgeons, the sick were all kept in the orlop deck.

On the 28th of September, the combined fleets left Rogerwick, and on October the 4th we entered the harbour of Carlsrcona, and here another circumstance is observable, which further proves that the fatal diseases among the Swedes were dysentery and typhus, for if it had been scurvy, the sick would certainly have recovered by the vegetable food that was there offered to them; instead of which, greater numbers were taken ill, and the mortality was very much increased; the deaths on board the ships were from forty to fifty daily, and the mortality at the hospital was equally dreadful.

At this period, the physician of the Baltic fleet arrived, and advised, (which, indeed, was the only means of saving the unhappy sufferers,) that the healthy as well as the sick should be sent on shore, and should have a plentiful allowance of vegetables and fruit; but as this advice was only partially attended to, the deaths continued still to be very numerous. Dr. Jamison found that the prevailing disease was scurvy, but the sick did not die of symptoms peculiar to this disease, but were soon attacked by dysentery or typhus, which shortly proved fatal.

On the whole, my opinion of the Swedish sailors was this, they were men worn out by fatigue and bad food, despondent and scorbutic, entirely prædisposed to receive any contagious disease that might be presented to them, at which time fever and dysentery made their appearance; and as no means were taken to prevent the spreading of the contagion, it rapidly spread itself over the different ships, and gave rise to all the devastation that afterwards prevailed when the ships went into port; but even then, although the sick were increased in number, although some of the medical attendants were seized by the complaint, yet I heard it alleged that it was not believed to be contagious, because *it depended upon scurvy*; and in *proof* of this, it was stated that it did not spread among the towns people.* To this I may answer, that the inhabitants of Carlscrona had not the prædisposition to the disease, and at any rate the communication between the hospital and the town is very small, and Carlscrona is a place not disposed to spread contagion, the streets being very wide, and the houses separate.

We left Carlscrona Oct. 21st. and here I must mention a splendid instance of worthiness in the English physician, who, commiserating the miserable condition of the Swedes, offered to remain with them, and take them under his own charge; but this offer, one of the most amicable that one government could make to another, was not accepted, I understand, through the means of the Swedish King's physician, who came down to examine into the state of the navy.

I have nothing more to add, except a few remarks on the treatment of these contagious diseases as they appear in

* I had an opportunity of hearing to day, (Nov. 17) that the Swedes continue to suffer at Carlscrona, and that Dr. Jamison, the English physician of the Baltic fleet, had been sent for to take charge of them; so that I have no doubt that a speedy stop will be put to the fatality, by the advice of so eminent a man, and I scarcely doubt but that he will use means similar to those I have mentioned above.

in a scorbutic person, which certainly must in some measure be regulated by the preceding disease; hence a vegetable diet becomes necessary, and particularly acid fruits should be liberally offered; in one case a pint or more of lime juice was given daily and the patient recovered, so that the citric acid, in *this case,* as well as in genuine scurvy, may form an excellent remedy, and may be preferable to the other acids that are sometimes used in this complaint. In this respect the Swedes seem to have acted very unfortunately, for they made their patients swallow large quantities of nitre and vinegar.

I may further add, that in the Swedish navy still greater means should be taken to avert scurvy than in the English, for most of the men are taken from the different estates of the commanders, where they have been employed as husbandmen, and they are always paid off on the approach of winter; but instead of this, the government allows them no lime juice, or any substitute for it, nor are the men allowed any beds, so that they are often half naked and lie upon the bare hammocks, and hence the numerous cases of rheumatism that continually prevail among them.

I am, &c.

JOSEPH ARNOLD.

*Nov.* 18, 1808.

